# The Medical Student, or Aids to the Study of Medicine

**Published:** 1844-12

**Authors:** 


Art. V.—*The Medical Student, or Aids to the Study of Medicine.* A revised and modified edition. By Robley Dunglison, M.D., Professor of the Institutes of Medicine, etc., etc. Philadelphia: Lea & Blanchard. 1844. 12mo. pp. 311.

Much of this work has been before the public, for some time, in the shape of *introductory lectures;* but the profession will be glad to receive it in its present modified and improved form. It is called the *Medical Student*, but it addresses itself equally to the medical *Teacher*, by whom it is greatly to be wished, that its matured opinions and counsel may be gravely pondered. "The whole of the observations," says the author, "apply to the study of medicine as taught in this country." They are such as reach all classes of teachers, and apply to the profession in private stations, as well as to those engaged in public instruction. The topics discussed are numerous, and the argument is continually illustrated and enforced by apposite and pleasing biographical sketches of eminent medical men. Thus, the first chapter, on "*preliminary education*," closes with notices of John Hunter, Armstrong, Good, and Godman, as affording, in their rise to the first eminence in their profession, evidence of what may be accomplished by persevering industry. In this chapter the author, very sensibly, as we think, counsels to the banishment of Latin *diplomas*, and Latin *prescriptions.* Very ludicrous examples of bad grammar, and confusion of tongues in the latter, are given; as in the following:

Recipe.—*Nitrate of potass*, sesquidrachmam.
*Tartrite of antimony*, granum.
Calomelanos præparati, grana quatuor.
Misce. Divide in octo (partes.)

This, for its jumble of languages, is equal to Dr. Caustic's motto of Greek, English, and Latin:

*O anthropos, or, ė gune,*
*Lacessit,* never, *me impune.*

The author says, truly:

"Perhaps, after all, one great cause of the continuance of the present mode of writing prescriptions is, that veneration for antiquity, which vindicates the Latin as the language for diplomas. The whole form of the prescriptiou is, indeed, a memento of by-gone periods, when Jove was invoked for his blessing on the medicine, and when symbols—unknown except to the initiated—were always employed. Medicine was then an 'art or mystery;' and the prescription of the physician equally with the labels on the bottles and boxes of the apothecary, conveyed the idea of that mystery, which has been properly designated as imperfect knowledge; but now, that this has been discarded,—that the arcana of the science are thrown freely open, and that the darkness and complicated dogmas of the schools have yielded to a better mode of reasoning and experiment, so that what was formerly taught and implicitly credited, as a dictum of the master, is now exhibited perspicuously and demonstratively, and, unless rendered intrinsically clear and intelligible, is unhesitatingly rejected,—these relics of a barbarous period ought to be discarded."

"Medical education prior to attendance on lectures" is the subject of the second chapter. This contains a pretty full glossary of technical terms, and Latin phrases, which will be acceptable to many medical students. The chapter closes with an answer to the question, what are the subjects which the office-student should peruse during his first year, and before he has commenced his attendance on lectures? The author remarks, that—

"Generally, on this point, the preceptor gives himself but little trouble. The youth is received into the office: the books,—few or many as the case may be—are placed at his disposal, and he is left to his own discretion—which may be a negative quantity—as to the topics he shall persue. Under such circumstances, it will almost always happen, that those subjects, which minister most to his curiosity, and which are, therefore, the least dry, will first attract his attention, and a discursive habit may, in this manner, be acquired, which may shed its injurious influence over his subsequent career."

"During the first year of office study, *full* benefit cannot accrue from the perusal of works on any of the branches of medical science. Perhaps, the most proper to be placed in the student's hands would be a treatise on physiology, which contains sufficient anatomy to enable him to acquire the terms, and to have a general idea of the structure and functions of the different parts of the organism. If he possess but a slight acquaintance with chemistry, general anatomy or the anatomy of the textures can be studied, at this period, almost as well as at any other."

Our author quotes, with approbation, the following remarks of a learned author, Dr. Latham, on the propensity of men to magnify the difficulties which the student must overcome, before making himself master of his profession:

"The different professions"—observes a recent learned author, Dr. Latham—"have one way of glorifying themselves, which is common to all. It is by setting forth a vast array of preparatory studies, and pretending they are indispensable in order to fit a man for the simple exercise of the practical duties that belong to them. I have heard lawyers make such a mighty parade of the things, which a man must know before he is called to the bar, that, according to the average of human capacities, not one in fifty has the smallest chance of mastering them; and of those who do master them, not one in fifty can employ them to the uses for which they are intended. I once saw a list of books recommended by a professor of divinity to the study of those going into holy orders. They were more numerous than the majority of even studious men ever read in their whole lives; yet these were a few prolegomena introductory to the office of a parish priest. We, too, conceive that it befits our dignity to magnify ourselves at certain seasons. The commencement of a session is usually the time chosen; and then, what a crowd of wonderful things are marshalled by authority around the entrance of our profession! And through this crowd, it is implied, every man must press his way before he can obtain admission. As if we wished to guard and garrison ourselves against invaders, rather than to gain good and useful confederates! In the affair of literature are reckoned Latin, and Greek, and French, and Italian, and German. In the affair of science, mathematics, and metaphysics, and mechanics, and optics, and hydraulics, and pneumatics, mineralogy, botany, zoology, and geology. Such are the portentous forms that guard the threshold. But farther onward are placed anatomy,—human and comparative, and morbid; physiology and pathology; chemistry,—general and pharmaceutical, and materia medica; surgery,—theoretical, clinical, operative, and ophthalmical; medicine,—theoretical, clinical, obstetrical, and forensical. The general display of objects so grand and multifarious is formidable enough; but not so formidable as their representation in detail. Of the great cosmogony of medicine there are several departments, and each professor never fails to magnify his own, by counting the cost of time and labour, which you must be prepared to bestow, if you wish to make any progress in it. "Haller (perhaps such an one will say) surely knew what anatomy is, and how much goes to make an atatomist; and Haller has estimated the cost at twenty years of time and labour."

The following passage, from the next chapter, is amusing and instructive. Having detailed some of the follies of the "*Brandy and Salt*" practice, by which mercenary men deceived the public, and put money in their purses, he adds:

"Yet, the more sad and solemn part remains. We find the possessor of these views, which seem to us so irrational, patronized not only by the long-suffering, capricious, and confiding female, but by men, who, in the pursuit of their own honest daily avocations, exhibit no lack of good sense; and by others, who, from their opportunities and position, ought to be expected to reject unhesitatingly such marvellous insignificancies; and who, on other subjects, exert a judicious skepticism, and a just appreciation of ordinary events."

And this grave reflection he illustrates by the capital anecdote which follows:

"It is entirely consistent with the manifestations of the human mind, that excessive credulity and excessive skepticism should exist at the same time in the same person; and that one who is a declared infidel on many topics that are admitted by the wisest and the best, may yet cherish the marvellous and the monstrous. The ancient but apposite anecdote of the flying-fish is, doubtless, known to many; but it will bear repetition, and has been presented again, of late, by a popular writer, C. Mackay, on a congenerous subject. 'Well, son John,' said the old woman, 'and what wonderful things did you meet with all the time that you were at sea?' 'Oh!

mother,' replted John, 'I saw many strange things.' 'Tell us all about them,' replied his mother, 'for I long to hear your adventures.' 'Well, then,' said John,—'as we were sailing over the line, what do you think we saw?' 'I can't imagine,' replied his mother. 'Well, we saw a fish rise out of the sea, and fly over our ship!' 'O, John, John! what a liar you are!' said his mother, shaking her head, and smiling incredulously. 'True as death!' said John; 'and we saw stili more wonderful things than that.' 'Let us hear them,' said his mother, shaking her head again; 'and tell the truth, John, if you can.' 'Believe it or believe it not, as you please,' replied her son; 'but as we were sailing up the Red Sea, our Captain thought he would like some fish for dinner, so he told us to throw our nets and catch some.' 'Well?' inquired his mother, seeing that he paused in his story. 'Well,' rejoined her son, 'we did throw them, and, at the very first haul, we brought up a chariot-wheel, made all of gold, and inlaid with diamonds!' 'Lord bless us!' said his mother; 'and what did the Captain say?' 'Why he said it was one of the wheels of Pharaoh's chariot, that had lain in the Red Sea ever since that wicked king was drowned, with all his host, whilst pursuing the Israelites.' 'Well, well!' said his mother, lifting up her hands in admiration, 'now that's very possible, and I think the captain was a very sensible man. Tell me such stories as that, and I'll believe you; but never talk to me of such things as flying-fish! No, no, John! such stories won't go down with me, I can assure you."

The concluding chapter, on medical education after graduation, contains judicious advice to the young practitioner, the substance of which may be guessed at from the heads of of the topics They are as follows: Graduation not the end of Study; Dignity of the profession; Advice to the graduate; Mode of observing; Fallacies of experience; Residence in an Hospital advised; Necessity for the exercise of sympathy and benevolence, and of attending to the address; Importance of temperance and sobriety, and of presence of mind; Obligations to secrecy, discretion and honor; Value of tact as well as talent; Professional etiquette; Qualifications of the young physician.

This volume will find interested readers in the numerous physicians who have been pupils of the author. But we trust its perusal will not be confined to them. It is a seasonable contribution to our medical literature, and will introduce an improved system of study among the profession, if its precepts are duly heeded. Y.

